# Drug-interactive mPEG-*b*-PLA-Phe(Boc) micelles enhance the tolerance and anti-tumor efficacy of docetaxel

**DOI:** 10.1080/10717544.2020.1718245

**Published:** 2020-01-31

**Authors:** Feirong Gong, Rongrong Wang, Zhengquan Zhu, Jiayao Duan, Xin Teng, Zhong-Kai Cui

**Affiliations:** a Key Laboratory for Ultrafine Materials of Ministry of Education, School of Materials Science and Engineering, East China University of Science and Technology, Shanghai, China;; b Department of Cell Biology, School of Basic Medical Sciences, Southern Medical University, Guangzhou, China;; c Department of Otorhinolaryngology, The Third Affiliated Hospital, Southern Medical University, Guangzhou, China;; d Guangzhou Regenerative Medicine and Health Guangdong Laboratory, Guangzhou, China

**Keywords:** Polymeric micelles, docetaxel, anti-tumor efficacy

## Abstract

Docetaxel (DTX) is one of the most promising chemotherapeutic agents for a variety of solid tumors. However, the clinical efficacy of the marketed formulation, Taxotere^®^, is limited due to its poor aqueous solubility, side effects caused by the emulsifier, and low selective DTX distribution *in vivo*. Here a facile, well-defined, and easy-to-scale up DTX-loaded *N*-(tert-butoxycarbonyl)-_L_-phenylalanine end-capped methoxy-poly(ethylene glycol)-*block*-poly(_D,L_-lactide) (mPEG-*b*-PLA-Phe(Boc)) micelles (DTX-PMs) were prepared in an effort to develop a less toxic and more efficacious docetaxel formulation. The physicochemical properties, pharmacokinetics, biodistribution, and *in vivo* anti-tumor efficacy were evaluated in comparison to the marketed DTX formulation Taxotere^®^. DTX was successfully encapsulated in the hydrophobic micellar core with a high encapsulation efficiency (> 95%) and a high drug loading capacity (4.81 ± 0.08%). DTX-PMs exhibited outstanding stability in the aqueous environment due to the strong interactions between the terminal amino acid residues and docetaxel. The pharmacokinetic study in Sprague–Dawley rats revealed higher DTX concentrations in both whole blood and plasma for the group treated with DTX-PMs than that treated with Taxotere^®^ due to the improved stability of the micellar formulation. In human non-small cell lung cancer (A549) tumor-bearing Balb/c nude mice, DTX-PMs significantly improved DTX accumulation and stalled DTX elimination in tumors than in bone marrow. Furthermore, only by half of the DTX dosage, our DTX/mPEG-b-PLA-Phe(Boc) micelles can achieve similar therapeutic effects as Taxotere^®^. Altogether, DTX-PMs hold great promise as a simple and effective drug delivery system for cancer chemotherapy.

## 1. Introduction

Docetaxel (DTX), an analog of paclitaxel, is an anti-cancer reagent of the taxoid family. Compared with paclitaxel, DTX exhibits a better affinity to tubulin and higher anti-tumor bioactivity. DTX has been approved by the Food and Drug Administration (FDA) for the treatment of various solid tumors such as locally advanced and metastatic breast cancer, non-small cell lung cancer, androgen-independent prostate cancer, and advanced gastric cancers (Gao et al., 2008; Liu et al., 2008, 2016). However, because of the poor aqueous solubility (6–7 μg/mL) of DTX, Tween-80 and ethanol have to be added in its marketed formulation (Taxotere^®^) as the emulsifier, resulting in undesirable side effects, including short-lasting neurotoxicity, hypersensitivity, fluid retention, hemolysis, and alopecia (Balakrishnan et al., 2016).

Various formulations of DTX have been prepared to enhance its solubility, reduce the adverse effects, and improve the efficacy (Immordino et al., 2003; Yin et al., 2009; Sanna et al., 2011; Luo et al., 2018). One of the most promising approaches was to encapsulate DTX in polymeric micelles. Compared to low-molecular-weight surfactant micelles, i.e. Tween-80, with a high critical micelle concentration (CMC) value of >100 mg/L, amphiphilic polymers form stable micelles with lower CMC values ranging between 10 and 100 mg/L to improve not only the solubility but also the pharmacokinetics and tumor selectivity of the encapsulated drugs via the enhanced permeability and retention (EPR) effect (Wang et al., 2015; Gothwal et al., 2016).

Although many amphiphilic copolymers can form micellar structures, and many types of micelles have been designed and investigated for DTX delivery, only a few of them have entered the clinical stage and Taxotere^®^ is still the only DTX formulation approved by the FDA. For micelle forming materials, diblock copolymers based on poly(ethylene glycol) (PEG), poly(_D,L_-lactide) (PLA), poly(glycolic acid) (PGA), and poly(ε-caprolactone) (PCL) are the most acknowledged candidates owing to their readily tailorable chemical structures, high drug loading capacity, ease for scale-up preparation, and biocompatibility in humans (Cavallaro et al., 2015; Cho et al., 2016). However, amphiphilic block copolymers based on them, such as PEG-*b*-PLA, present a few critical problems when they were explored in DTX formulations (Gaucher et al., 2010). First, most of them were not sufficiently stable to retain the encapsulated drug in the tumor site via the EPR effect after administration. Second, the *in vivo* anti-tumor bioactivity of such micellar DTX formulations was not significantly improved. Therefore, it is exigent to explore novel polymeric micelles that are stable to deliver DTX to tumor sites and afford higher anti-tumor efficacy.

Previously, we have conjugated N-(tert-butoxycarbonyl)-_L_-phenylalanine (Boc-_L_-Phe) to the terminal hydroxyl group of methoxy-poly(ethylene glycol)-*block*-poly(lactide) (mPEG-*b*-PLA) and yielded an end-capped block copolymer, mPEG-*b*-PLA-Phe(Boc) (Han et al., 2016). Carbazitaxel encapsulated micelles formed by this copolymer showed significantly higher stability than carbazitaxel-loaded mPEG-*b*-PLA micelles, as that the Boc-_L_-Phe and drugs could interact through π–π stacking, hydrogen bonding, and hydrophobic force (Han et al., 2016). To improve the stability and anti-tumor efficacy, DTX was encapsulated in mPEG-*b*-PLA-Phe(Boc) micelles (DTX-PMs) in this study in an effort to develop a less toxic and more efficacious docetaxel formulation. The physicochemical properties of DTX-PMs including size distribution, encapsulation efficiency, *in vitro* stability, and release profile of DTX were characterized and their cytotoxicity, pharmacokinetics, and anti-tumor bioactivity were further evaluated to demonstrate the potential as a DTX delivery system.

## 2. Experimental section

### 2.1. Materials

**Figure 1.**
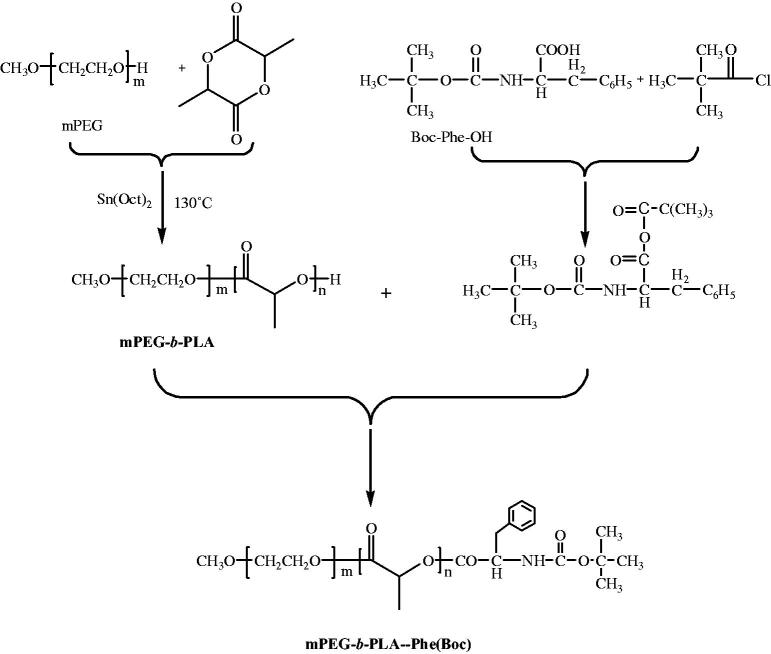
Synthesis route of mPEG-*b*-PLA-Phe(Boc).

mPEG-*b*-PLA-Phe(Boc) with a number-average molecular weight (M_n_) of 3300 g/mol and a polydispersity index (PDI) of 1.03 was synthesized according to our previous protocol (Gong et al., 2017). The chemical structure and synthetic route of mPEG-*b*-PLA-Phe(Boc) are shown in Figure 1. Docetaxel anhydrous was purchased from Xingyinhe Chemical Inc. (Wuhan, China). Taxotere^®^ (Sanofi-Aventis, Aventis Pharma; Frankfurt, Germany) was purchased and stored in dark at 4 °C. 3-(4,5-dimethyl-2-thiazolyl)-2-5-diphenyl tetrazolium bromide (MTT) purchased from Sigma-Aldrich (Milwaukee, WI, USA) was diluted in 0.01 M phosphate-buffered saline (PBS) (pH = 7.4) at 5 mg/mL then stored at −20 °C. Other chemicals were of reagent grade and used as received.

### 2.2. Cell line and animals

Human non-small cell lung cancer (NSCLC) cell lines (NCI-H460 and A549) were a gift from Prof Ping Shi in the School of Biotechnology at East China University of Science and Technology. The cells were cultured in Roswell Park Memorial Institute (RPMI) 1640 medium (Invitrogen GmbH, Karlsruhe, Germany) containing 10% heat-inactivated fetal bovine serum (FBS) and 1% antibiotics (100 U/mL penicillin and 100 U/mL streptomycin) (Burlington, ON, Canada) at 37 °C in a humidified 5% CO_2_ atmosphere.

Male and female specific pathogen-free (SPF) Sprague–Dawley rats (250 ± 5 g) and Balb/c nude mice (21 ± 2 g, 4–6 weeks old) were purchased from Vital River (Beijing, China). All animals were housed in a room maintained at 23 ± 3 °C, 65–75% humidity, with a controlled 12 h light–dark cycle for 5–7 days before experiments. Water and commercial laboratory complete food for animals were available *ad libitum*. All animal procedures were conducted following the protocol approved by the Institutional Animal Care and Use Committee of Mabspace Biosciences Co. (Suzhou, China).

### 2.3. Preparation of docetaxel-loaded polymeric micelles

DTX-PMs were prepared using a solid dispersion-thin film hydration method (Muley et al., 2016). Briefly, DTX (20 mg) and mPEG-*b*-PLA-Phe(Boc) (380 mg) were dissolved in 5 mL of ethanol at 45 °C. Ethanol was then rotatory evaporated under reduced pressure to obtain a polymer matrix containing DTX. Then 5 mL of ultrapure water was added and the mixture was stirred gently at 45 °C until a transparent clear DTX-PMs dispersion was formed. D-mannitol (80 mg) was added to the micelle dispersion as the cryoprotectant. Then the micellar dispersion was filtered through a 0.22 µm PVDF filter (Millipore, Shanghai, China) to eliminate non-encapsulated drug and distributed in sterile vials before lyophilization. As a comparison, DTX-loaded mPEG-*b*-PLA micelles (DTX/mPEG-*b*-PLA micelles) were prepared following the same protocol except that mPEG-*b*-PLA-Phe(Boc) was replaced by mPEG-*b*-PLA. Blank micelles were prepared following the same protocol in the absence of DTX. Various concentrations of DTX encapsulated in mPEG-PLA-Phe(Boc) and mPEG-PLA micelles were prepared for evaluation of the *in vitro* stability at 37 °C.

### 2.4. Particle size and morphology

The mean particle size and size distribution of the micelles were measured using a Zetasizer Nano Dynamic Light Scattering (DLS) apparatus (Malvern Instruments, Malvern, UK) at a scattering angle of 173°. Each freeze-dried sample was reconstituted in 5 mL of ultrapure water and 1 mL of the dispersion was placed in a plastic cuvette for the measurement. Data acquisition was carried out using the automatic adjustment of position, attenuation, and measurement time. Three measurements were performed to obtain the *Z*-average hydrodynamic diameter along with the standard deviation, and the PDI was obtained from the ratio of the second-order cumulant divided by the square of the first-order one. The results were expressed as mean size ± standard deviation for three independent experiments.

The morphology of the micelles was observed using a JEM-2100 transmission electron microscope (TEM, JEOL, Tokyo, Japan) at an acceleration voltage of 75 kV (Mikhail & Allen, 2010). Reconstituted micellar dispersions were dropped on copper grids covered with carbon film then negatively stained with 2.0% sodium phosphotungstate, providing an electron-dense layer to obtain reverse-contrast, negative-electron images. Samples were deposited on copper grids and briefly left to evaporate the solvent prior to the observation.

### 2.5. Drug-loading efficiency

The amount of DTX encapsulated in the DTX-PMs was measured using Agilent 1260 high-performance liquid chromatography (HPLC) (Agilent Technologies, Santa Clara, CA, USA). The lyophilized micelles were reconstituted in ultrapure water and then centrifuged at 12,400 *g* for 15 min to eliminate non-encapsulated drug. The amount of DTX loaded in the micelles in the supernatant was measured using HPLC with a reverse-phase HPLC column (Agilent Eclipse XDB-C18, 4.6 mm × 250 mm, 5 μm) (Muthu et al., 2015). Briefly, 0.1 mL of the micellar dispersion was dissolved in 10 mL of the mobile phase that consisted of HPLC-grade acetonitrile and ultrapure water (55:45, *v/v*) for micellar disruption. The solution was filtered through a 0.45 μm syringe filter and the filtrate was stored in a HPLC vial. An aliquot (20 μL) of the sample was injected into HPLC and detected with a UV/Vis detector at 230 nm at the elution flow rate of 1 mL/min. The drug encapsulation efficiency (EE, %) was calculated by dividing the actual amount of drug in micelles by the total amount of drug in solution. The drug loading capacity (DLC, %) was calculated by dividing the weight of drug in micelles by the weight of micelles.

### 2.6. Stability of reconstituted micelles *in vitro*

Lyophilized DTX-PMs and DTX/mPEG-*b*-PLA micelles were reconstituted in saline and incubated at 4 and 25 °C, in which the initial concentration of DTX was 4 mg/mL. The released DTX crystalized and subsequently precipitated in the dispersion because of its low aqueous solubility. At predetermined time points, appropriate amount of dispersion was centrifuged at 12,400 g for 15 min to remove the precipitate. Then the actual concentration of DTX in the supernatant was determined following the same HPLC protocol described above. Appearance of the micellar dispersion was observed and recorded in the meantime.

### 2.7. *In vitro* DTX release profile

A dynamic dialysis method was performed to investigate the *in vitro* release behavior of DTX. A presoaked dialysis membrane tube (M_W_ cutoff: 8000–14,000 g·mol^-1^) added with the DTX-PM dispersion (1 mL) was placed in 200 mL PBS buffer (0.05 M, pH 7.4) that contained 0.2 % (*w/v*) Tween-80, followed by incubation with shaking at 37 ± 0.5 °C and 100 rpm. Dissolution medium (1 mL) was collected at predetermined time points and replenished with an isochoric fresh medium to maintain a constant volume. The amount of DTX released was determined following the same HPLC protocol described in Section 2.5. The cumulative release was expressed as the total percentage of DTX released over time. The DTX release behaviors of Taxotere^®^ and DTX/mPEG-*b*-PLA micelles were also determined following the same protocol.

### 2.8. *In vitro* cytotoxicity

The cytotoxic effect of the solvent of Taxotere^®^ (s-Taxotere, 50/50 *v/v* Tween-80 and 13% ethanol), blank PMs, Taxotere^®^, and DTX-PMs against NCI-H460 cells were evaluated using the MTT assay. Briefly, NCI-H460 cells were cultured in 96-well plates at 1 × 10^4^ cells/well in 100 μL culture medium and incubated for 24 h. Then dilutions of various formulations in 100 μL complete medium were added to the wells. After 48 h, 10 μL of MTT solution (5 mg/mL in 0.01 M PBS, pH 7.4) was added and incubated for another 4 h. Then cell culture medium was replaced with 100 µL of dimethylsulfoxide. The absorbance at the wavelength (*λ*) of 570 nm was measured on a SpectraMax 190 Molecular Device (Molecular Devices Corporation, Sunnyvale, CA, USA) removing background absorbance at *λ* of 655 nm. The cell viability (%) was calculated by normalizing the absorbance of the treated cells exposed to different formulations by the value of the control, untreated cells. NCI-H460 cells were selected for cytotoxicity study *in vitro* because they proliferated faster and they are more sensitive to DTX than the A549 cells.

### 2.9. Pharmacokinetics in healthy animal model

Male and female SPF grade Sprague–Dawley rats, six for each group, weighing 250 ± 5 g, were used for the pharmacokinetic studies. Polyethylene cannulas were implanted into the jugular vein of rats for blood sample collection. Rats were housed in metabolism cages. Blood samples were drawn the day before the experiment as the control. The DTX-PMs or Taxotere^®^ at the DTX dose of 10 mg/kg were injected via the tail vein. Blood samples were collected from the canthus at predetermined time points (0.083, 0.25, 0.5, 1, 3, 8, and 24 h) and centrifuged at 3500 *g* for 5 min for further analysis. An LC-MS/MS protocol was established and validated to determine the DTX concentration in blood plasma using a LC-MS 2020 system equipped with a UV–visible detector (Shimadzu, Tokyo, Japan) and the detection limit of the equipment was 1 ng/g. Chromatography was performed using a 5 μm Phenomenex Phenyl-Hexyl column (100 mm length × 2.0 mm diameter) at 50 °C at a flow rate of 0.5 mL/min. The initial elute was 5 mM ammonium formate in water/acetonitrile (60:40). A 0.6-min, linear gradient to 5 mM formic acid in acetonitrile was initiated, followed by a 2.5-min hold before re-equilibrating the column in the initial mobile phase for 4 min. The total LC effluent was directed to waste from 0 to 3.0 min post-sample injection and then introduced into a Sciex API 3000 triple quadruple mass spectrometer between 3.0 and 6.4 min. Ionization was achieved using turbo ion spray in the positive ion mode. The linear range was 2–2000 ng/mL. Pharmacokinetic parameters were assessed using non-compartmental pharmacokinetic methods with the DAS software (version 2.0, Beijing, China).

### 2.10. Pharmacokinetics and biodistribution in A549 xenograft tumor model

Human NSCLC cell line A549 was grown in the culture medium as described above. The cells in the logarithmic phase were digested with a 0.05% trypsin solution and re-suspended in the cell culture medium. After centrifugation at 200 *g* for 5 min, cell pellets were re-suspended in PBS. Female Balb/c nude mice were implanted subcutaneously (s.c.) on the mid-right side with 8 × 10^6^ cells in 0.1 mL PBS (84 mice). When the heteroplastic A549 tumor reached a volume of approximately 200 mm^3^, mice were divided randomly into two groups and seven subgroups in either group with six mice per subgroup. One group received Taxotere^®^ and the other received DTX-PMs at a single DTX dose of 10 mg/kg. Mice in one subgroup were sacrificed and then the blood, bone marrow, and tumor samples were collected at 0.083, 0.25, 0.5, 1, 3, 8, and 24 h (*n* = 6), respectively, post-administration. Samples were dissolved in tert-butyl methyl ether and the concentration of DTX was determined by validated LC-MS/MS method described above.

### 2.11. Anti-tumor efficacy

Human NSCLC cell line A549 cells were cultured as described above. Balb/c nude mice were implanted s.c. on the mid-right side with 1 × 10^7^ A549 cells in 0.2 mL PBS (64 mice in each xenograft model) (Gonzalez-Fajardo et al., 2016). When the heteroplastic A549 tumor reached a volume of ∼150 mm^3^, mice were divided randomly into eight groups with eight mice per group. Group 1 (G1) received physiological saline as the negative control. Group 2 (G2) received blank PMs. Groups 3, 4, and 5 (G3, G4, and G5) were treated with Taxotere^®^ at DTX doses of 2.5, 5, and 10 mg/kg, respectively. Groups 6, 7, and 8 (G6, G7, and G8) were injected with DTX-PMs at DTX doses of 2.5, 5, and 10 mg/kg, respectively. All the mice were injected via the tail vein once every four days for three treatments (Q4D × 3). Mice were weighed and tumors were measured individually with a Vernier caliper every 4 days. The tumor volume was calculated using the equation of length × width^2^/2. The tumor inhibitory rate (%) was calculated using the following equation, (*W*_c_ − *W*_t_)/*W*_c_, where *W*_c_ is the weight of the tumor in the control group and *W*_t_ is the weight of the tumor in the experimental group.

### 2.12. Statistical analysis

Data are expressed as mean value ± standard deviation. Statistical significance was assessed using the one-way analysis of variance (ANOVA). The analysis of variances followed by Tukey’s post hoc test was employed in this work with SPSS13.0 (SPSS, Chicago, IL, USA). For all statistical comparisons, the difference with *p*<.05 was considered statistically significant.

## 3. Results and discussion

### 3.1. Preparation and characterization of DTX-PMs

DTX-PMs could be easily prepared using a facile thin-film hydration method. Table 1 presents the physicochemical characterizations of blank and DTX-loaded mPEG-PLA-Phe(Boc) and mPEG-PLA micelles. Hydrophobic DTX interacted with the hydrophobic block of the copolymer and was trapped in the micellar core. Although no significant difference in drug loading capacity between mPEG-PLA and mPEG-PLA-Phe(Boc) micelles was observed, the stability of DTX/mPEG-PLA-Phe(Boc) micelles (DTX-PMs) at 37 °C was much higher than that of DTX/mPEG-PLA micelles. In addition, the hydrodynamic size of DTX-PMs micelles was evidently smaller than that of DTX/mPEG-PLA micelles, indicating much stronger interactions between DTX and the amino acid in mPEG-PLA-Phe(Boc) than the terminal hydroxyl group in mPEG-PLA. DTX could be formulated in mPEG-PLA-Phe(Boc) micelles at a high drug feed ratio of 7.5%, however, the resulting DTX-PMs only remained stable for 2 h at 37 °C. An increase in the polymer/drug feed ratio was significantly correlated with the colloidal stability of the micelles. Therefore, the drug feed ratio of 5 % was selected for the preparation of micelles in the following experiments.

**Table 1.** Physicochemical characterizations of blank and DTX-loaded mPEG-PLA and mPEG-PLA-Phe(Boc) micelles at 37 °C.

Micelles	Feed drug ratio	Size (nm)	PDI	^a^Stability (h)
mPEG-PLA	–	20.3 ± 0.42	0.091	–
DTX/mPEG-PLA	7.5	27.6 ± 0.56	0.089	<0.5
	5.0	25.8 ± 0.64	0.078	<1
	2.5	23.6 ± 0.23	0.056	<2
mPEG-PLA-Phe(Boc)	–	23.4 ± 0.64	0.094	–
DTX/mPEG-PLA-Phe(Boc)	7.5	18.7 ± 0.38	0.075	<2
	5.0	17.4 ± 0.58	0.062	>24
	2.5	17.2 ± 0.33	0.047	>72

a Stability: time of precipitation appeared.

**Figure 2.**
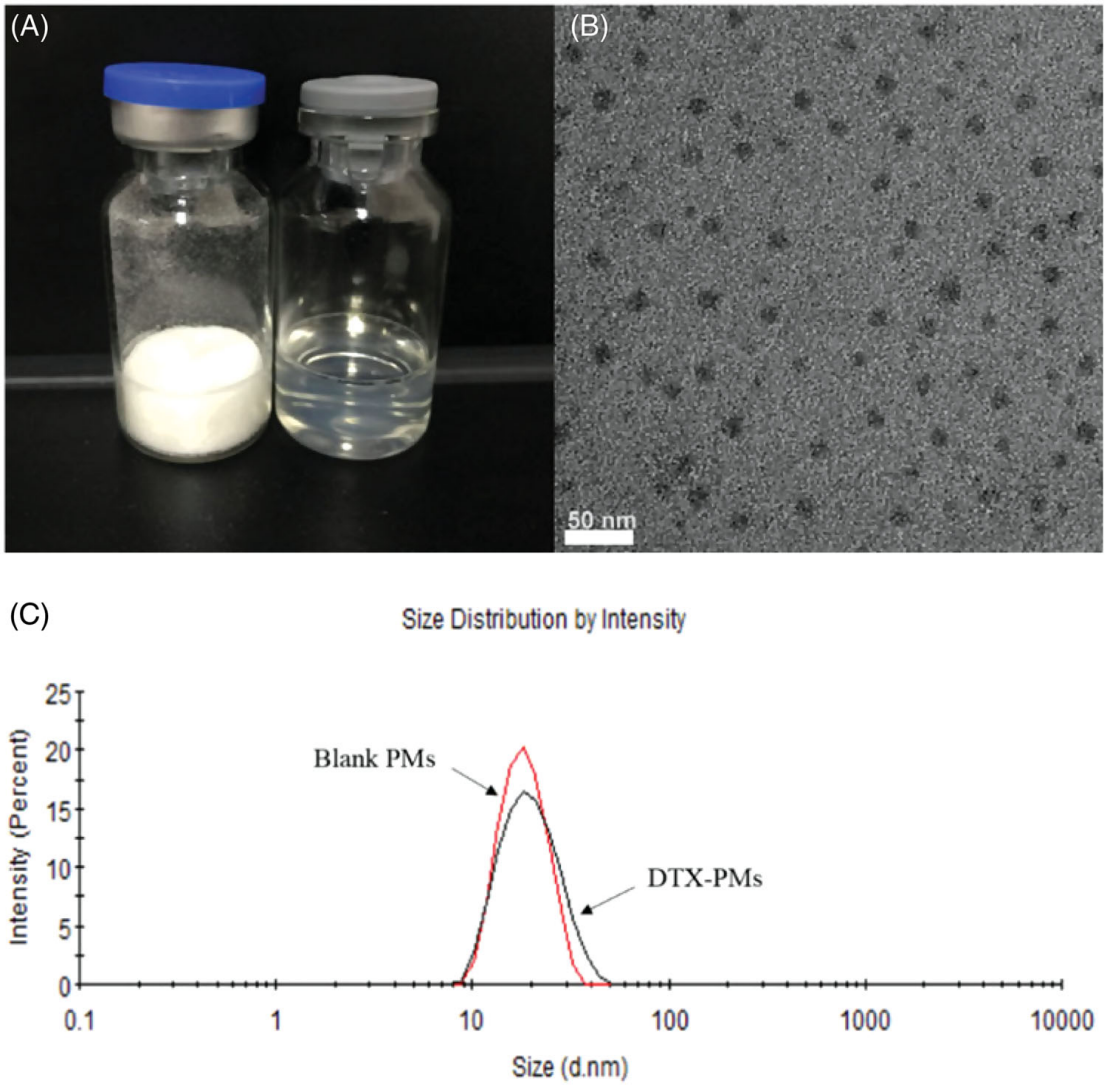
Appearance of the freeze-dried DTX-PMs (left vial) and reconstituted dispersion in water (right vial) (A); morphology of DTX-PMs observed using TEM (B); size distribution of blank PMs and DTX-PMs determined using DLS (C).

As shown in Figure 2(A), lyophilized micelles were uniform, white solid (left vial) and easy to reconstitute in aqueous media such as pure water and saline by simply shaking to form uniform transparent dispersion (right vial). The TEM photograph in Figure 2(B) shows that the micelles had a regular spherical shape with a monodisperse distribution. As shown in Figure 2(C), the hydrodynamic diameter of micelles was typically ∼20 nm and the particle size exhibited a narrow distribution with a monodisperse unimodal pattern, as shown in Figure 1(B). Encapsulation of DTX in the micelles rarely affected the micellar diameter and size distribution. When the theoretical DLC was 5%, the EE and actual DLC of DTX-PMs were 95.97 ± 0.02% and 4.81 ± 0.08%, respectively. In consequence, the aqueous solubility of DTX was markedly improved from 0.006 to more than 10 mg/mL by a factor of ∼1600. When reconstituted in saline for injection, DTX-PMs exhibited outstanding stability in the aqueous environment due to the strong interactions between Boc-_L_-Phe and docetaxel, including π − π stacking, hydrogen bonding, and hydrophobic force compared with Taxotere^®^. DTX-PMs dispersion was dynamically stable for at least 72 h at room temperature without showing any precipitation, which was significantly more stable than DTX/mPEG-*b*-PLA micelles (Lee et al., 2011).

**Figure 3.**
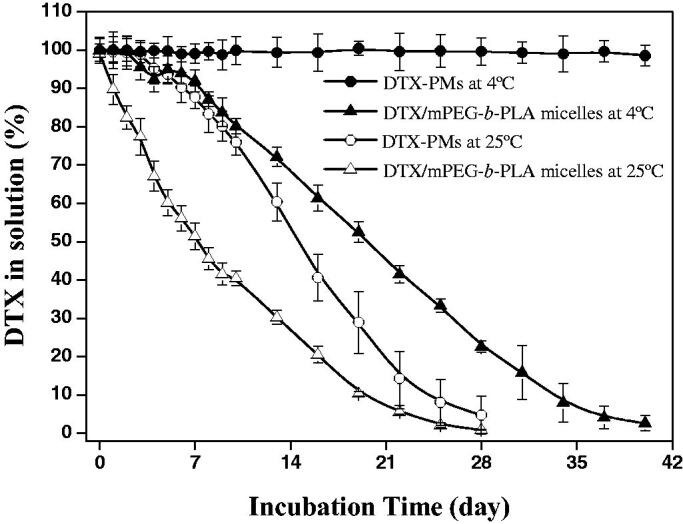
Storage stability of DTX-micelles. Percentage of DTX solubilized in PBS/Tween-80 (0.01 M/0.2%, pH 7.4) versus incubation time at 4 and 25 °C.

The storage stability of DTX-PMs was evaluated. As shown in Figure 3, the concentration of DTX in the dispersion began to decrease at 72 h but remained more than 85% even after one week at 25 °C. When incubated at 4 °C, the reconstituted dispersion of DTX-PMs was clear and transparent, and the concentration of DTX loaded in the micelles was always close to the initial value of 4 mg/mL for more than 40 days. In contrast, DTX/mPEG-*b*-PLA micelles tended to aggregate, and the micelle dispersion became muddy within 6 h at room temperature. DTX in the dispersion greatly decreased and was less than 85% of the initial value at 48 h and dropped to 51.4% of the initial value after one week. The storage stability was better at 4 °C but still much lower than that of DTX-PMs. The DTX in the dispersion of DTX/mPEG-*b*-PLA micelles was less than 70% after 2 weeks and almost all the DTX released after 40 days. The results showed that the introduction of Boc-_L_-Phe enhanced the interactions between the micellar core and DTX, therefore improved the stability of the micelles. A previous work from our laboratory showed that incorporation of Boc-_L_-Phe at the end of mPEG-*b*-PCL could reinforce carrier–carrier and carrier–drug interactions by hydrophobic force, hydrogen bonding, and π − π stacking of aromatic rings, leading to a more tightly packed micelle core (Gong et al., 2017).

### 3.2. *In vitro* DTX release profile

**Figure 4.**
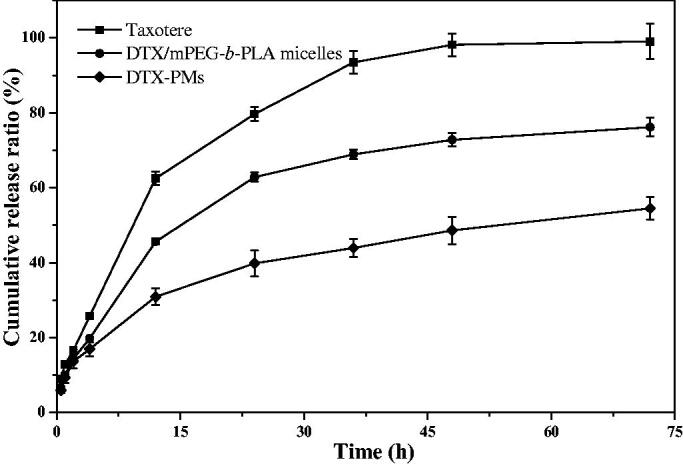
*In vitro* DTX release profile.

The *in vitro* release behavior of DTX from Taxotere^®^, DTX/mPEG-*b*-PLA micelles, and DTX-PMs was investigated using the dynamic dialysis method. As shown in Figure 4, the cumulative DTX release from Taxotere^®^ and DTX/mPEG-*b*-PLA micelles was more than that from DTX-PMs at the same time point. After 12 h, 62.5% DTX released from Taxotere^®^, and 45.6% DTX released from DTX/mPEG-*b*-PLA micelles, whereas only 30.9% DTX released cumulatively from DTX-PMs. Almost all the DTX released from Taxotere^®^ at 48 h, 72.8% DTX released from DTX/mPEG-*b*-PLA micelles, while less than 50% DTX released from DTX-PMs. Drug release from micellar formulations involves drug diffusion and micelles disassemble (Kim & Park 2010), which was a relatively slow process. The slower drug release from DTX-PMs than that from DTX/mPEG-b-PLA micelles further verified the stability of DTX-PMs improved through the interactions between Boc-_L_-Phe and the drug molecule.

### 3.3. *In vitro* cytotoxicity

**Figure 5.**
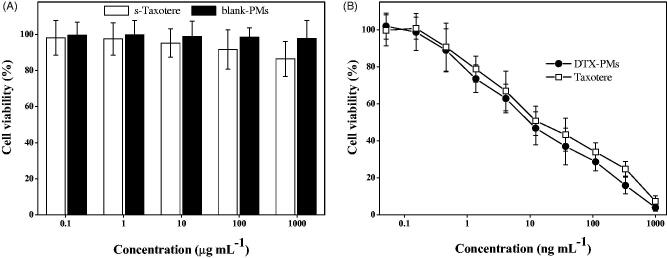
*In vitro* cytotoxicity of the emulsifier of Taxotere^®^ (50/50 *v/v* Tween-80 and 13% ethanol), blank PMs, Taxotere^®^, and DTX-PMs against NCI-H460 cells.

The *in vitro* cytotoxicity of DTX-PMs was evaluated using NCI-H460 cells and compared with that of Taxotere^®^. As shown in Figure 5(A), blank PMs exhibited negligible cytotoxicity in the investigated period and NCI-H460 cell viability was nearly 100% even at a high concentration of 1000 µg/mL. The viability of the cells exposed to the emulsifier of Taxotere^®^, i.e. Tween-80 at 500 µg/mL and 13% ethanol at 500 µg/mL, decreased to 85%, which was lower than that of blank PMs. For drug-loaded formulations, both DTX-PMs and Taxotere^®^ presented evidently cytotoxicity in a similar manner and the cell viability decreased significantly in a dose-dependent manner (Figure 5(B)). The 50% inhibitory concentrations (IC_50_) for Taxotere^®^ and DTX-PMs were 15.1 ± 0.05 and 11.2 ± 0.05 ng/mL, respectively. This result indicated that the mPEG-*b*-PLA-Phe(Boc) micellar formulation could effectively deliver DTX to cancer cells.

### 3.4. Pharmacokinetics in healthy animal model

**Figure 6.**
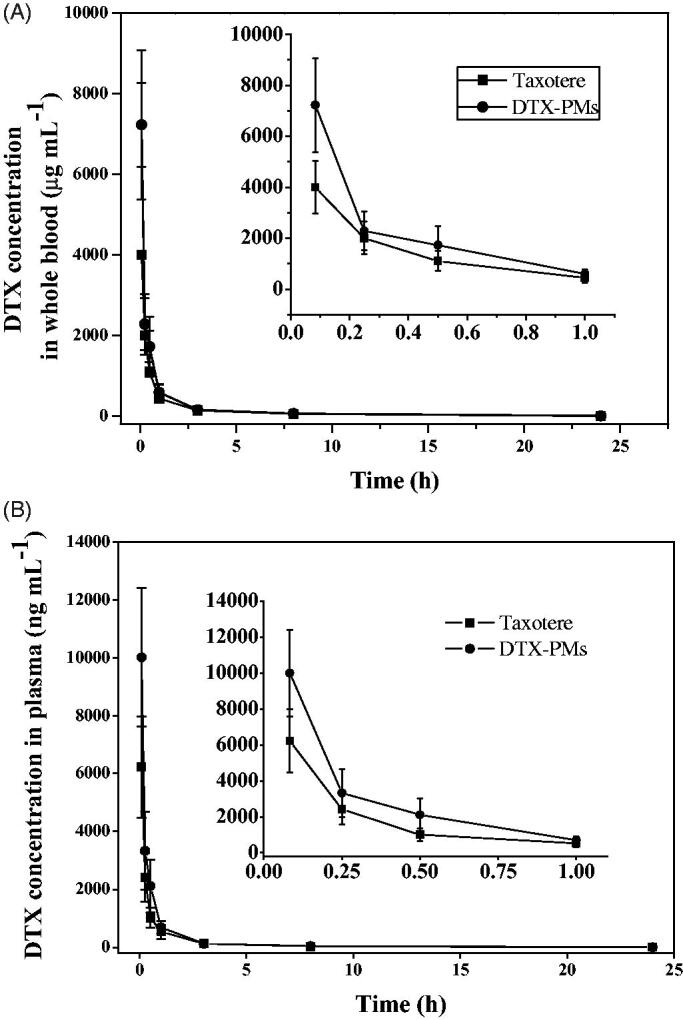
DTX distribution of Taxotere^®^ and DTX-PMs in whole blood (A) and plasma (B) of Sprague–Dawley rats after i.v. administration at a single dose of 10 mg/kg.

The pharmacokinetic profiles of DTX-PMs and Taxotere^®^ after i.v. administration are shown in Figure 6 and the calculated pharmacokinetic parameters are listed in Table 2. K_el_ was the elimination rate constant for DTX according to the laws of the first-order reaction kinetics, *t*_1/2_ is the elimination half-life of DTX and is calculated using 0.693/K_el_, *t*_max_ is the time to reach the maximum concentration (*C*_max_) and CL was the clearance of DTX from the body. The variation of the DTX concentration after *i.v.* administration of DTX-PMs was similar to that of Taxotere^®^, while the DTX level in the group treated with DTX-PMs was always higher than the latter, both in the whole blood (Figure 6(A)) and in the plasma (Figure 6(B)). The area under the curve (AUC) showed that DTX exposure of DTX-PMs was significantly higher than that of Taxotere^®^ in plasma (*p*<.05). The apparent volume of distribution calculated using the steady-state method (Vd_ss_) of DTX from DTX-PMs was evidently lower than that from Taxotere^®^ in the whole blood (*p*<.05). Those results revealed that DTX administrated in the form of DTX-PMs partitioned slower from plasma to tissues compared with DTX delivered by Taxotere^®^, which might result from the improved stability through micelle encapsulation of the drug. Furthermore, *C*_max_ and mean residence time (MRT_IV_) of DTX-PMs were significantly higher (*p*<.05) than the values of Taxotere^®^ both in the whole blood and plasma. The half-life of DTX in the DTX-PMs was reduced slightly in the whole blood compared with that in Taxotere^®^.

**Table 2.** Pharmacokinetic parameters for Taxotere^®^ and DTX-PMs in Sprague–Dawley rats following *i.v.* administration (10 mg DTX/kg).

Parameters	Unit	Whole blood			Plasma		
		Taxotere^®^	DTX-PMs	*p*	Taxotere^®^	DTX-PMs	*p*
K_el_	h^−1^	0.0394	0.0519	*.024	0.0366	0.0399	.149
*t*_1/2_	h	18	14	*.032	19	18	.151
*C*_max_	ng·mL^−1^	1503	2185	*.005	1225	2224	*.002
AUC_0–t_	ng·h·mL^−1^	4313	4407	.785	1470	2170	*.007
AUC_0–inf_	ng·h·mL^−1^	4973	4742	.508	1541	2236	*.002
AUMC_0–t_	ng·h^2^·mL^−1^	49509	39240	*.021	6959	7376	.471
AUMC_0–inf_	ng·h^2^·mL^−1^	99127	62151	*.000	12356	12262	.945
CL	mL·min^−1^·kg^−1^	33.5	35.9	.327	109	76.4	*.000
MRT_IV_	h	20	13	*.000	8.0	5.5	*.001
Vd_SS_	L·kg^−1^	40.1	28.5	*.008	52.0	25.3	*.000

* *p*<.05.

### 3.5. Pharmacokinetics and biodistribution in xenograft tumor model

**Figure 7.**
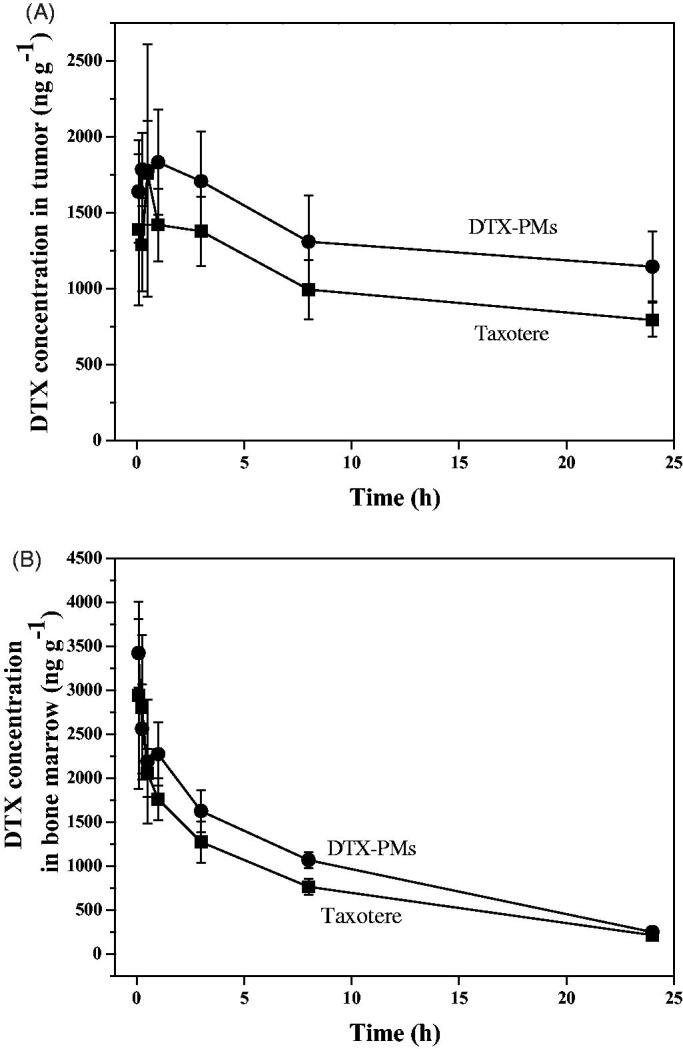
DTX distribution of Taxotere^®^ and DTX-PMs in tumor (A) and bone marrow (B) of A549 xenograft Balb/c nude mice model after i.v. administration at a single dose of 10 mg/kg.

The pharmacokinetic profiles of DTX-PMs and Taxotere^®^ in A549 xenograft tumor model after i.v. administration are illustrated in Figure 7, and the calculated pharmacokinetic parameters are listed in Table 3. The DTX-PMs showed a significantly higher DTX concentration in tumor than Taxotere^®^ (Figure 7(A)), and the drug exposure level (AUC_0–inf_) of DTX-PMs in the tumor was 1.8 times more than that of Taxotere^®^. On the other hand, no significant difference of the DTX concentration in bone marrow, the toxic target tissue of DTX, was observed between DTX-PMs and Taxotere^®^ (Figure 7(B)). The ratio of AUC_0–inf_ between bone marrow and plasma of Taxotere^®^ and DTX-PMs was 5.67 and 4.96, respectively, indicating that drug exposure of micellar DTX in bone marrow was lower than that of the commercial formulation. The ratio of drug exposure levels (AUC_0–inf_) between tumor and plasma of Taxotere^®^ and DTX-PMs were 15.40 and 19.26, respectively, indicating that the relative drug exposure in the tumor, the pharmacodynamic target tissue of DTX, of DTX-PMs was significantly higher (*p*<.05) than that of Taxotere^®^. In addition, the *t*_max_ and MRT of DTX-PMs in the tumor were evidently longer than that of Taxotere^®^ (*p*<.05), suggesting that DTX-PMs were a sustained-release formulation and had longer drug retention time in tumor than Taxotere^®^. Therefore, DTX-PMs were more tumor-targeted in tumor-bearing mice and presented higher drug potency than Taxotere^®^. Collectively, the DTX-PM formulation was more effective than Taxotere^®^ in this *in vivo* setting.

**Table 3.** Pharmacokinetic parameters for Taxotere^®^ and DTX-PMs in A549 xenograft tumor Balb/c nude mice following i.v. administration (10 mg DTX/kg).

Parameters	Unit	Whole blood		Plasma		Bone marrow		Tumor	
		Taxotere^®^	DTX-PMs	Taxotere^®^	DTX-PMs	Taxotere^®^	DTX-PMs	Taxotere^®^	DTX-PMs
K_el_	h^−1^	0.135	0.128	0.144	0.143	0.0838	0.0898	0.0251	0.0164
*t*_1/2_	h	5.2	5.4	4.8	4.9	8.3	7.7	28	42*
*t*_max_	h					0.083	0.083	0.5	1.0*
*C*_max_	ng·g^−1^	3999	7230*	6226	10,011*	2942	3423	1763	1835
AUC_0–t_	h·ng·g^−1^	3293	4694	3605	5267	18,095	23,518	24,525	32,443
AUC_0–inf_	h·ng·g^−1^	3353	4775	3642	5306*	20,636	26,292	56,223	102,198*
AUMC_0–t_	h·h·ng·g^−1^	8822	10,866*	6299	7617	121,116	157,943	252,972	350,754
AUMC_0–inf_	h·h·ng·g^−1^	10,688	13,441*	7439	8834	212,418	255,420	2,277,576	6,270,774*
MRT	h	3.2	2.8	2.0	1.7	10	9.7	41	61*

* *p*<.05.

### 3.6. *In vivo* anti-tumor efficacy against A549 human NSCLC xenograft model

As reviewed in the World Cancer Report 2014, lung cancer is the main cause of cancer-related deaths (Stewart & Wild, 2014). In the present study, human NSCLC A549 tumor xenograft model was selected to verify the anti-tumor efficacy of DTX-PMs.

**Figure 8.**
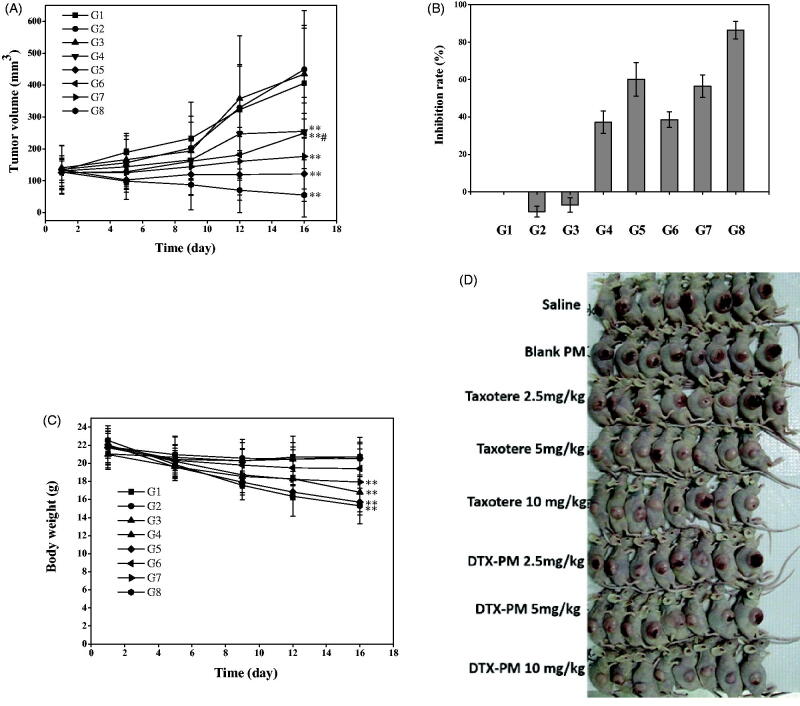
*In vivo* anti-tumor efficacy in A549 xenograft model. G1, saline; G2, blank PMs; G3-5, Taxotere^®^ at DTX doses of 2.5, 5, and 10 mg/kg; G6–8: DTX-PMs at DTX doses of 2.5, 5, and 10 mg/kg. (A) Tumor growth curves (tumor volume versus time); (B) tumor inhibition rate; (C) body weight; (D) images of mice with A549 tumors. Each data point is mean ± standard deviation (*n* = 8). **p<.01 versus control (saline); *p<.05 versus control (saline); ^#^*p*<.05 versus Taxotere^®^.

During the treatment of the A549 tumor-bearing mice, the tumor volumes were measured at days 1, 5, 9, 12, and 16, and at last, the whole tumors were excised and weighed at day 16. Figure 8(A) shows tumor volume evolution during the treatment period. The tumors in three DTX-PMs groups showed growth retardation compared with that in the control group after the second treatment. The tumor size in the groups treated with DTX-PMs was smaller than that in the Taxotere^®^-treated groups at the same dose. The low (2.5 mg/kg, G3) and medium (5 mg/kg, G4) doses of Taxotere^®^ could hardly inhibit the growth of tumor (*p*>.05), while the same dose of DTX-PMs (G6 and G7) stalled tumor growth significantly (*p<*.05). Taxotere^®^, even at a high dose of DTX (10 mg/kg, G5), exhibited only moderate anti-tumor efficacy and resulted in a tumor volume of 121.4 mm^3^ at the end of the treatment. In contrast, the tumor volume in the group treated with DTX-PMs was only 55.2 mm^3^, suggesting that DTX-PMs formulation was an effective approach to inhibiting tumor growth in the A549 model. As shown in Figure 8(B), DTX-PMs exhibited a higher inhibition rate than Taxotere^®^, indicating that DTX-PMs could inhibit tumor growth more effectively. The inhibitory effect of DTX at high dose (10 mg/kg, G5) in Taxotere^®^ was 60.12%, while that of DTX-PMs (G8) was significantly higher (86.41%). It is worth noting that the treatment efficacy was similar for G5 and G7, although the DTX dose in the G7 group treated with DTX-PMs was only half of that in the G5 group treated with Taxotere^®^. This result can be attributed to the enhanced uptake of the drug by the tumor tissues through the EPR effect. In comparison with the saline control group, the bodyweight of mice (Figure 8(C)) in both groups treated with Taxotere^®^ and DTX-PMs decreased, in particular, the percentage of body weight changes in G5 and G8 was both higher than 20% with no significant difference at day 16, while acceptable body weight losses (13%) occurred in G3, G4, G6, and G7 during the treatment. This result indicated that i.v. administration of DTX-PMs at 5 mg/kg induced similar anti-tumor efficacy compared with Taxotere^®^ at 10 mg/kg in A549 human NSCLC xenograft model. Besides, the group treated with blank PMs showed no difference in body weight change from the control group, suggesting no intrinsic toxicity of the micelles.

Moreover, throughout the entire treatment period, no toxicity-induced death was observed in any experimental group. The two DTX formulations were effective in preventing tumor growth compared with saline, while DTX-PM was found to be more efficacious than Taxotere^®^. These results clearly indicated that DTX-PMs could be more efficacious for chemotherapy by reducing the dose of anti-cancer drugs to dilute or even avoid undesirable adverse effects.

## 4. Conclusions

DTX-PMs showed remarkably enhanced aqueous solubility of DTX by a factor of ∼1600, which could circumvent the toxicity of the commercially used surfactant Tween-80 in Taxotere^^®^^. The obtained micellar formulation showed outstanding drug-loaded micelle stability in the aqueous dispersion *in vitro*, beneficial for convenient transportation and clinical usage. The pharmacokinetics study revealed that DTX administrated in the form of PMs partitioned slower from plasma to tissues compared with DTX delivered using Taxotere^®^ as the result of the improved stability of the micellar formulation. The pharmacokinetics and distribution of DTX-PMs in A549 xenograft tumor model showed that DTX-PM was a tumor targeted and sustained-release formulation with higher drug potency than Taxotere^®^. The *in vivo* xenograft studies revealed that the DTX-PM was more effective in inhibiting tumor growth and more biocompatible to mice compared with Taxotere^®^, consistent with the results of the *in vitro* cytotoxicity assay. Thus, DTX-PMs hold high potential as a water-based formulation of DTX with enhanced anti-tumor bioactivity to treat disparate tumors. A better understanding of the internalization and fate of DTX-PMs inside tumor cells and the incorporation of other biologically relevant cleavable functional groups in the polymer structure are under our investigation to improve the *in vivo* performance of the formulation.
